# Infection strategies of mycoplasmas: Unraveling the panoply of virulence factors

**DOI:** 10.1080/21505594.2021.1889813

**Published:** 2021-03-11

**Authors:** Chen Yiwen, Wu Yueyue, Qin Lianmei, Zhu Cuiming, You Xiaoxing

**Affiliations:** Institute of Pathogenic Biology, Hengyang Medical College, University of South China; Hunan Provincial Key Laboratory for Special Pathogens Prevention and Control; Hunan Province Cooperative Innovation Center for Molecular Target New Drug Study, Hengyang, China

**Keywords:** Mycoplasmas, adhesins, invasive enzymes, metabolites, toxins

## Abstract

Mycoplasmas, the smallest bacteria lacking a cell wall, can cause various diseases in both humans and animals. Mycoplasmas harbor a variety of virulence factors that enable them to overcome numerous barriers of entry into the host; using accessory proteins, mycoplasma adhesins can bind to the receptors or extracellular matrix of the host cell. Although the host immune system can eradicate the invading mycoplasma in most cases, a few sagacious mycoplasmas employ a series of invasion and immune escape strategies to ensure their continued survival within their hosts. For instance, capsular polysaccharides are crucial for anti-phagocytosis and immunomodulation. Invasive enzymes degrade reactive oxygen species, neutrophil extracellular traps, and immunoglobulins. Biofilm formation is important for establishing a persistent infection. During proliferation, successfully surviving mycoplasmas generate numerous metabolites, including hydrogen peroxide, ammonia and hydrogen sulfide; or secrete various exotoxins, such as community-acquired respiratory distress syndrome toxin, and hemolysins; and express various pathogenic enzymes, all of which have potent toxic effects on host cells. Furthermore, some inherent components of mycoplasmas, such as lipids, membrane lipoproteins, and even mycoplasma-generated superantigens, can exert a significant pathogenic impact on the host cells or the immune system. In this review, we describe the proposed virulence factors in the toolkit of notorious mycoplasmas to better understand the pathogenic features of these bacteria, along with their pathogenic mechanisms.

## INTRODUCTION

**Figure 1.**
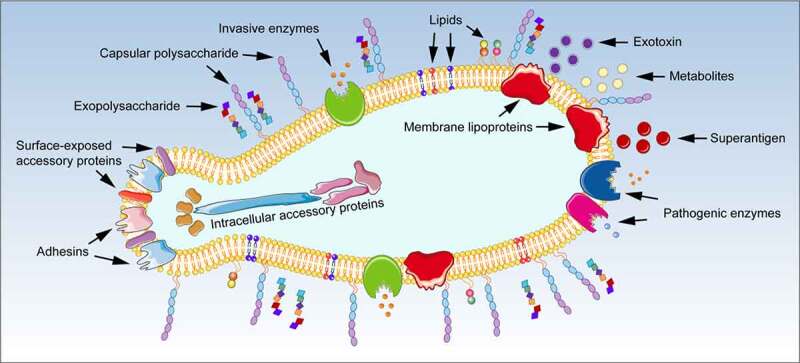
**Schematic diagram of virulence factors in mycoplasmas**. Virulence factors in mycoplasmas include invasiveness, toxin-like substances, exotoxins, pathogenic enzymes, and some membrane components. Invasiveness refers to the ability of mycoplasmas to break through the host’s defense function and to settle, reproduce, and spread *in vivo*; it is mediated by various factors that comprise adhesins and accessory proteins, capsular polysaccharides, invasive enzymes, and biofilms. Toxin-like substances primarily include metabolites generated during the process of proliferation, such as H_2_O_2_, NH_3,_ and H_2_S. Certain mycoplasmas can also secrete some exotoxins including CARDS TX and hemolysin, as well as express various pathogenic enzymes, such as lipolytic enzymes, peptidases, phosphatases, ecto-ATPases, cytotoxic nucleases and nucleotidases, which are considered important pathogenic factors for mycoplasmas. In addition, some inherent molecules of the cell membrane such as lipids, membrane lipoproteins, and the superantigen produced by *M. arthritidis*, may also have a significant pathogenic effect on the host cells or the immune system

Mycoplasmas were first described 100 years ago. To date more than 210 species have been identified to be widely distributed among humans, animals, insects, and plants. Unlike gram-negative bacteria, mycoplasmas are the smallest and simplest self-replicating bacteria. Mycoplasmas, lack a rigid cell wall, use the universal stop codon UGA for tryptophan, and are small enough to pass through bacterial-retaining filters. Mycoplasmas are classified into the Mollicutes class [1,2]. Most human and animal mollicutes belong to the *Mycoplasma* and *Ureaplasma* genera of the family Mycoplasmataceae. Mycoplasmas evolved from gram-positive bacteria that underwent significant genome reduction; as such, most members of this genus exhibit host and tissue specificities and have limited metabolic options for replication and survival, forcing them to adapt to procure metabolic precursors from the host [3]. To accomplish this pathogenic process, mycoplasmas must first overcome several obstacles to successfully invade the host’s defense and reach full lifecycles. The invasiveness of mycoplasmas is mediated by surface adhesins and their accessory proteins, capsular polysaccharides, invasive enzymes, and biofilms, all of which ensure their establishment, reproduction, and spread *in vivo* [4–7]. During the parasitism cycle, mycoplasmas absorb nutrients from host cells and release a large number of metabolites, such as hydrogen peroxide (H_2_O_2_), ammonia (NH_3_) and hydrogen sulfide (H_2_S), causing localized tissue damage [8,9]. Certain mycoplasmas can also secrete some exotoxins, including community-acquired respiratory distress syndrome toxin (CARDS TX), and hemolysins [10,11]. These toxins have different functions and can affect host cell functions in several ways. Mycoplasmas can also express various pathogenic enzymes such as lipolytic enzymes, peptidases, phosphatases, ecto-ATPases, cytotoxic nucleases and nucleotidases, that are considered important mycoplasma pathogenic factors [12,13]. Furthermore, some inherent components located at the mycoplasma cell membrane, such as lipids, membrane lipoproteins, and even superantigens produced by *Mycoplasma arthritidis*, could trigger an inflammatory response through various strategies (Figure 1) [14–16]. Although several questions remain unanswered, significant progress has been made in identifying the virulence factors by which mycoplasmas interact with and subsequently damage the host cells. In this review, we aim to discuss the primary virulence factors of mycoplasmas to better understand the strategies used to penetrate the circulation system and increase infectivity in both humans and animals.

## INVASIVENESS OF MYCOPLASMAS

### Adhesins

Adhesion is an initial and essential step for subsequent colonization and infection, and the loss of adhesion activity corresponds to a great decrease in pathogenicity for most mycoplasmas [1]. In a narrow sense, the cell components that demonstrate direct cytadhesion are collectively called adhesins, whereas those involved in the adhesion process that do not directly interact with host cell substances are referred to as accessory proteins.

#### Adhesins for pathogenic mycoplasmas in human

The most studied mycoplasmas possessing adhesins are *M. pneumoniae* and *M. genitalium*. The common characteristic of these two mycoplasmas is the formation of a flask-shaped and filamentous tipped structure known as terminal organelle. The most predominant adhesins in *M. pneumoniae* are P1 and P30 [17]. P1 is a remarkably versatile molecule that forms a complex with P30, P40, and P90; these colocalize to the tip of the terminal organelle to perform different functions, such as receptor recognition and gliding motility [4,18]. Schmitt et al. has found that the P1 complex also contains P65, DnaK, C-terminal truncated forms of DnaK and P1, pyruvate dehydrogenase E1 α subunit (PDHA), HMW1, and HMW3 to coordinate the adherence of *M. pneumoniae* to host cells [4,19]. The P30 adhesin, originally regarded as an accessory protein, plays a role in localizing P1 to the terminal organelle and is involved in cell development [18]; the amino acid sequence of P30 holds substantial homology with some eukaryotic proteins in humans, indicating that P30 could be associated with the occurrence of certain autoimmune diseases [18]. P1 and P30 are highly immunoreactive; monoclonal anti-P1/P30 antibodies can inhibit cytadherence while the lack of P1/P30 protein(s) causes no adhesion [20]. Recently, the immunoreactive protein P116 was also regarded as an *M. pneumoniae* adhesin. Similar to P1, P116 is sensitive to trypsin [18]. However, the exact function of the P116 adhesin warrants further investigation.

Upon cytadherence initiation, the adhesins require a stable primary association with certain biological macromolecules on the host tissues; the extracellular matrix (ECM) is a typical example of these macromolecules and includes fibronectin (Fn), plasminogen (Plg), heparin, fibrinogen, vitronectin, mucin, sialylated molecules, oligosaccharides, glycolipids, and glycoproteins [4]. The well-characterized ECM-binding adhesins for *M. pneumoniae* are elongation factor thermo-unstable (EF-Tu) and glyceraldehyde-3-phosphate dehydrogenase (GAPDH) [17,21]. The carboxyl regions of EF-Tu and the amino acids 340 to 358 within the Fn-binding region 2 are critical for Fn-*M. pneumoniae* interactions [22]. However, anti-GAPDH serum did not affect the adherence of *M. pneumoniae* to HeLa cells [23], suggesting that GAPDH has a limited influence on adhesion. Similarly, the pyruvate dehydrogenase E1 β subunit (PDHB) of *M. pneumoniae* is a Fn- and Plg-binding adhesin [21,24]. Sialylated molecules are a class of host targets discovered in recent years that can bind to mycoplasma adhesins, such as *M. pneumoniae* P1 and P30, *M. genitalium* P110 and ureaplasmas [25–27]. Certain surface-exposed glycolytic enzymes of mycoplasmas, have recently been recognized as adhesins that participate in pathogen-host interactions. For example, *M. pneumoniae* contains nine glycolytic enzymes, including alpha-enolase (Eno), pyruvate kinase (PK), GAPDH, pyruvate dehydrogenases A–C, lactate dehydrogenase, phosphoglycerate mutase, and transketolase, all of which interact with some ECM components, such as Fn, Plg, fibrinogen, vitronectin, lactoferrin, and laminin, to play a potential role in *M. pneumoniae* adherence and invasion [24,28,29]. Recently, the chaperones DanK and GroEL of *M. pneumoniae* were demonstrated to combine with ECM [30], indicating that the chaperones are multifunctional and participate in the process of adhesion and dissemination in *M. pneumoniae* infections. Few typical ECM-binding adhesins are listed in Table 1.

**Table 1.** Characteristics of some established ECM-binding adhesins

Species	Adhesin	Molecular weight (KDa)	Antigenic variation	Fn	Plg	Heparin	References
*M. pneumoniae*	P1^a^	169	+	+	+	-	[4,20,87,95]
	EF-Tu	45	-	+	-	+	[21,22,88]
	GAPDH^a,c^	36.8	-	+	+	-	[23,89]
	PDHB^c^	36	-	+	+	-	[21,24]
	Eno	49.2	-	-	+	-	[24]
	DanK^a,c^	65.1	-	+	+	-	[30]
	GroEL^a,c^	58.1	-	+	+	-	[30]
*M. hyopneumoniae*	P97	97	+	-	-	+	[90]
	P102	102	-	+	-	-	[91]
	P116	116	-	+	+	-	[43]
	P146	148.2	+	-	+	+	[42]
	P159	159	-	-	-	+	[41]
	P216	216	-	-	-	+	[40]
	Mhp107	104	-	+	+	+	[45]
	Mhp271	118.8	+	+	-	+	[44]
	Mhp683	135	+	-	-	+	[46]
	Eno	49.4	-	+	+	-	[54]
	EF-Tu	43.6	-	+	-	-	[47]
	GAPDH	Unknown	-	+	+	+	[48]
	FBA	44	-	+	-	-	[50]
	MHJ_0125	~40	-	-	+	+	[51]
	MHJ_0461	51.4	-	-	+	+	[52]
*M. bovis*	P27	27	-	+	-	-	[69]
	Eno	49	-	-	+	-	[70]
	TrmFO	48.8	-	+	-	-	[74]
	NOX	49	-	+	-	-	[75]
	FBA	31.4	-	+	+	-	[72,73]
*M. gallisepticum*	PlpA	158	-	+	-	-	[92]
	Hlp3	175	-	+	-	-	[92]
	PDH complex^b^	Unknown	-	-	+	-	[65]
	Eno	50	-	-	+	-	[61]
*M. suis*	Eno	59	-	-	+	-	[53]
*M. fermentans*	Eno	~50	-	-	+	-	[37]
*M. synoviae*	Eno	~53	-	+	+	-	[93]
*M. conjunctivae*	LppT	105	-	+	-	-	[83]

a. can also bind to fibrinogen, vitronectin

b. including PDHA and PDHB

c. can also adhere to lactoferrin and laminin

The major adhesins of *M. genitalium* include P140 (known as MgPa and MgpB) and P110 (MgpC). P140 and P110 are reciprocally stabilized and beneficial to cell adhesion, gliding motility, and terminal organelle formation and development [31]. Moreover, P140 and P110 are prone to antigen variation, which is correlated to optimization of adhesion, access to nutrients, survival in the host and escape from the host defense mechanisms [25,32]. The loss of either P140 or P110 protein also results in non-cytadherence and a hemadsorption-negative phenotype with the concomitant inability to develop the terminal organelle [31]. Furthermore, *M. genitalium* GAPDH has been shown to bind to mucin [33]. This is the first report to demonstrate that mucin is involved in adherence and motility, and this GAPDH-mucin recognition event can help to better understand the mechanism of adherence, colonization and pathogenicity.

Variable adherence-associated (Vaa) antigen is the key adhesin of *M. hominis*. The secondary structure of the Vaa antigen contains an alpha-helical structure with a coiled-coil region, which mediates the adherence of *M. hominis* to host cells [34]. P50t is a truncated form of the Vaa adhesin, which adheres to macrophages to evoke an immune response via the upregulation of Toll-like receptor (TLR) 2 expression [35]. *M. fermentans* surface lipoprotein P29 is an adhesin that mediates adhesion of *M. fermentans* to HeLa cells, and undergoes phase variation by surface masking, which is distinct from Vaa that is governed by protein expression [36]. In contrast, *M. fermentans* also express Eno, which take part in adherence and invasion [37].

#### Adhesins for pathogenic mycoplasmas in swine

*M. hyopneumoniae* adhesin P97 is a cilium adhesin that can undergo antigenic variation and is thus involved in evasion of the host immune response [38]. P68 is another *M. hyopneumoniae* cilium adhesin that mediates the occurrence of inflammatory response and apoptosis [39]. Wherease, recent documents indicated that some surface-exposed adhesins, such as P216, P159, P146, P116, Mhp271, Mhp107 and Mhp683, are generated through endoproteolytic cleavage of the P97/P102 paralog family, and all of which are ECM-binding adhesins [40–46]. The P146 cilium adhesin bind to Plg through the C-terminal lysine and arginine residues [42]. Moreover, the EF-Tu and GAPDH of *M. hyopneumoniae* are Fn-binding adhesins and the ciliary border of the airway is the target for EF-Tu adhesion [47,48]. With the gradual advancement in the study on mycoplasma pathogenicity, some multifunctional enzymes have been considered as adhesins, for example, *M. hyopneumoniae* expresses fructose-1, 6-bisphosphate aldolase (FBA) and L-lactate dehydrogenase [49]. The function of FBA is similar to that in *M. bovis*, including invasion and persistent infections [50]. Moreover, both MHJ_0125, a glutamyl aminopeptidase, and MHJ_0461, a leucine aminopeptidase, have moonlighting roles as adhesins on the surface of *M. hyopneumoniae*; MHJ_0461 also binds to foreign double-stranded DNA [51,52]. Eno in *M. hyopneumoniae* and *M. suis* also play crucial roles in adherence, invasion, and infections [53,54].

The hemotropic *M. suis* also expresses immunodominant adhesin MGS1 (now named GAPDH), which shares a high homology within GAPDH in *M. penetrans* and can interact with Band3 and glycophorin A in erythrocytes [55,56]. Likewise, the adhesion characteristics of erythrocyte-binding adhesin O-sialoglycoprotein endopeptidase (OSGEP) of *M. suis* is similar to that of GAPDH [56].

The variable lipoprotein (Vlp) family in *M. hyorhinis* consists of seven members: VlpA–G, all of which consist of three regions, among which regions II and III are of utmost significance [57,58]; region II is a major cytadhesion site with the consensus sequence SQQPGSG; region III determines the adhesion capability of Vlp family members; for example, the adhesion efficiency of region III with 0 and 3 copies of repeat unit is stronger than that with 12 copies, which suggests that the function of region II may be influenced by a longer region III [58].

#### Adhesins for pathogenic mycoplasmas in avian

There are two major adhesins in *M. gallisepticum*, MGC1 and MGC2. MGC1 shares higher homology levels with *M. pneumoniae* P1 and *M. genitalium* P140, whereas MGC2 is more homologous with *M. pneumoniae* P30 and *M. genitalium* P32 [59,60], indicating that the functions of these adhesins may be similar to those of adhesins in *M. pneumoniae* and *M. genitalium. M. gallisepticum* can also utilize glycolytic enzymes as adhesins including triosephosphate isomerase (Tpi), Eno and PK [61]. Pretreatment with specific antibodies can significantly abrogate *M. gallisepticum* adherence or survival *in vitro*, and similar adhesins have also been found in *M. synoviae* [62–64]. Intriguingly, the PDHA and PDHB complex of *M. gallisepticum* is deemed to be a Plg-binding protein and the combination of anti-PDHA and PDHB antisera can block the adherence to DF-1 cells [65]. Likewise, *M. gallisepticum* adhesins PlpA and HIp3 are Fn-binding protein. Apart from the adhesins listed above, there remain numerous adhesins in *M. gallisepticum* including GapA, CrmA, PvpA and pMGA1.2 [66,67]. These adhesins are involved in early colonization and systemic infection to varying degrees.

Like *M. pneumoniae* P1 and P30, *M. synoviae* variable lipoprotein hemagglutinin (VlhA) can also bind to sialylated molecules [68]. The most important characteristic of this VlhA-sialylated molecule binding process is the presence of sialoreceptor binding motif P-X-(BCAA)-X-F-X-(BCAA)-X-A-K-X-G in sialylated receptors [68], indicating that this distinct motif might help identify hypothetical adhesins and their sialoreceptors.

#### Adhesins for pathogenic mycoplasmas in cattle and goat

*M. bovis* P27 is an immunogenic Fn-binding protein that mediates invasion and infection. Anti-P27 antiserum only partially inhibits attachment [69], indicating that P27 exert synergistic effect with other adhesins. *M. bovis* also express Eno and its function is the same as Eno in *M. hyopneumoniae* [70,71]. Similar *Eno* gene was also found in the genome of *M. bovirhinis* [3]. Some multifunctional enzymes including methylenetetrahydrofolate-tRNA-(uracil-5-)-methyltransferase (TrmFO), NADH oxidase (NOX), and FBA also found in *M. bovis*. Among these, TrmFO and FBA are involved in cytadhesion to EBL cells [71–74]. NOX functions as a membrane-associated adhesin by interacting with amyloid precursor-like protein 2 on EBL cells [75]. Recently, *M. bovis* Mbov_0503 has been observed to bind tight junctions and cross the epithelial barrier [76]; hence, it may be a potential virulence factor for colonization. Other adhesions of *M. bovis* (P26, Vsps, and VpmaX) also participate in the adhesion and pathogenesis of *M. bovis* [77–79].

*M. agalactiae* adhesins include P40 and the familial adhesins Vpmas (VpmaU–Z), with the latter functioning in invasins [80,81]. The *M. conjunctivae* adhesin LppS exhibits high homology with the adhesins in *M. hyopeneumoniae*, such as P146, MHP1, P97, and ciliary adhesin [82]. LppS contains a characteristic serine-rich region and a proline-rich region. *M. conjunctivae* also possesses LppT, which is similar to the *M. hyopeneumoniae* membrane proteins P76 and P110 [82]. The function of LppT is to assist LppS and participate in adherence to lamb cells [82,83]. The primary adhesin of *M. mycoids subsp. mycoids* (Mmm) is P19, and its adhesion ability is significantly reduced after antibody treatment [84]. One adhesin-related gene (XDU01000267) and four PDH complex genes were found in the *M. capricolum subsp. capripneumoniae* (Mccp) M1601 genome, but their functions need further verification [85].

#### Adhesins for pathogenic mycoplasmas in murine

There are two adhesins in *M. arthritidis*, Maa1 and Maa2, both of which can induce protective immunity; however, monoclonal antibodies against Maa1 and Maa2 only partially inhibit cytadherence [86]. Maa1 is a major adhesin that is sensitive to trypsin, and its pathogenic role warrants further investigation. Interestingly, the *M. arthritidis* Maa2 mutant showed enhanced cytadherence [86]. However, the mechanism by which Maa2 regulates adhesion remains unclear and may be associated with epitope masking or “on/off”-switched phase variation.

Given the increasing identification of various adhesins in mycoplasmas and their associated functions functions, it remains challenging to reasonably classify these adhesins. In fact, many adhesins, such as some glycolytic molecules, have multiple properties and are also matrix-binding proteins. Even for the same adhesin, ectodomain shedding can occur under certain conditions, which results in functional diversity. For example, the P1 adhesin of *M. pneumoniae* can be subjected to post-translational processing, creating 22 proteoforms, each of which retains the ability to bind a variety of host molecules such as Fn, Plg, fibrinogen, vitronectin, and sialic acid; thus, the probability of *M. pneumoniae* infection is greatly increased [4]. Typically, there is more than one type of adhesin on the surface of mycoplasmas and a single adhesin may be insufficient for efficient adhesion; therefore, the cooperation of multiple molecules (including accessory proteins) is required to accomplish effective adhesion.

### Accessory proteins

Although certain proteins may not be directly involved in adhesion, their absence may cause some mycoplasmas to lose their adhesion ability; moreover, regaining their expression has been shown to restore cytadherence-positive phenotype [20]. These proteins are accessory proteins or adhesion-associated proteins, which play a crucial role in adherence, maintenance of the proper architecture of the terminal organelle, lateral movement, and anchoring of adhesins at the attachment organelle [18,31,94].

The most extensively studied mycoplasma for accessory proteins is *M. pneumoniae*, which incorporates two groups of accessory proteins, the first including P90 and P40, and the second including HMW1–3 and P65 [20,94]. P90 and P40 are surface-exposed proteins located in the terminal organelle near P1. Both P90 and P40 mutants result in P1 scattered on the cell surface rather than anchored at the tip structure [18,31,95,96]. In contrast, HMW1 and HMW3 are not located on the surface of *M. pneumoniae* but are involved in maintaining the morphology and stability of the tip structure, and in clustering other adhesins to this structure. HMW2 may be located at the base of the tip structure and is crucial to promote the stable and fully functional cell surface output of HMW1, HMW3, and P65 [1,18,97,98]. Furthermore, the loss of HMW1 also decreases the level of P65 [97]. However, the exact role of P65 remains unknown.

Another example of an accessory protein is TopJ, a J-domain chaperone molecule of *M. pneumoniae*, which is located at the base of the terminal organelle and is necessary for cytadhesion and gliding motility [99]. Interestingly, TopJ mutants maintain normal levels of P1, P30, P90, P40, HMW1, and HMW2, but their cytadhesion and motility are diminished, while the formation/positioning of terminal organelles is delayed or poorly coordinated with cell growth. Similarly, P1 fails to translocate or fold correctly on the surface of mycoplasma cells, indicating that TopJ may function in terminal organelle maturation, migration, or both, during cell division [99].

In addition to *M. pneumoniae*, certain accessory proteins, such as MG218 and MG317, were also found in *M. genitalium*, and are needed for adherence of P140 and P110 [100,101]. Beyond that, there were few reports on other mycoplasma accessory proteins have been published [102]. In general, if a gene mutation results in the loss of the adhesive feature, the encoded protein is likely to be preferentially considered as an adhesin. From this point of view, the boundary between adhesins and accessory proteins is becoming increasingly blurred. Therefore, it is necessary to define the explicit function of the putative adhesin prior to classifying it as an accessory protein.

### Capsular polysaccharides

A variety of mycoplasmas possess a layer of sticky capsule-like substance outside the cell membrane; chemical analysis indicates that its primary component is polysaccharide [103]. The formation of mycoplasma capsule is similar to that of the bacterial capsule. Generally, capsules are easily formed in the body but disappear *in vitro*; they are encoded by multiple genes [85], but there are exceptions. For example, only one capsule synthesis-related gene is annotated in the *M. bovirhinis* genome [3]. The formation of the capsule is attributed to glycosyltransferase, which is present in various mycoplasmas [103,104]. Mycoplasma capsular polysaccharide (CPS) is considered as an important virulence factor for pathogenic mycoplasmas as it is essential for adhesion, cell invasion, phase variation, and defense against immune systems such as anti-phagocytosis and anti-bacteriolytic activity [104–107].

Capsular polysaccharide plays an important role in the pathogenesis of mycoplasma. For example, *M. ovipneumoniae* CPS exerts a cytotoxic effect and induces apoptosis of sheep airway epithelial cells through a ROS-dependent JNK/P38 MAPK mechanism [108]; meanwhile, it can activate the TLR4-MyD88-NF-кB and TLR4-TRIF-IRF3 pathways to trigger overexpression of pro- and anti-inflammatory cytokines, such as IL-10 and TGF-β [106]. This anti-inflammatory cytokine-inducing activity is also observed in *M. pneumoniae* CPS and Mmm-derived free exopolysaccharide (EPS), which negatively regulates the immune system [109–111]. In addition, *M. pulmonis* secretes EPS-I, which is composed of galactose and glucose (ratio 1:1) and can be recognized by the lectin Griffonia simplicifolia I [105]. Like Mmm-derived polysaccharides, EPS-I can also offset the effects of the complement system [110,112–114]. Mutation of EPS-I results in decreased adherence to A549 and MLE-12 cells, an increased tendency for *M. pulmonis* to form biofilms on glass surfaces, and effective colonization of mouse lungs and trachea by overexpression of EPS-II [5,105,110,112,113], indicating that certain types of EPS may function in invasiveness. Moreover, certain capsules and polysaccharides are instrumental in facilitating the dissemination of mycoplasmas and in sustaining a chronic infection either by anti-phagocytosis or by downregulating the functions of macrophages [110,115–117]. Galactan, the galactofuranose homopolymer, serves as CPS and EPS in Mmm strains [117,118]. Similarly, the galactofuranose of CPS components is also a virulence factor in *M. mycoides subsp. capri* (Mmc) [110]. Furthermore, the galactofuranose in Mmm and Mmc is essential for membrane integrity and concealing adhesins but does not contribute to serum resistance [114].

### Invasive enzymes

Invasive enzymes are those synthesized by pathogenic bacteria during metabolism without destroying the host cells, while assisting in colonization, reproduction, and the spread of pathogenic bacteria. To date, several mycoplasmas have been identified to produce a variety of invasive enzymes, such as nucleases, immunoglobulin proteases, antioxidant enzymes, sialidases, and hyaluronidases. These enzymes play an important role in the mycoplasma growth and evasion of host immune surveillance. For a detailed introduction to these invasive enzymes, the readers can refer our previous review [119].

#### Nucleases

Nucleases are important pathogenic factors for certain mycoplasmas, which degrade host nucleic acids, and are used as a source of nucleotide precursors, thereby playing a pivotal role in growth, survival, persistence, and pathogenicity. The first nuclease was identified approximately 20 years ago. Since then, a number of similar enzymes or homologous genes have also been reported in *M. pneumoniae, M. genitalium, M. hominis, M. penetrans, M. bovis, M. agalactiae, M. meleagridis, M. gallisepticum, M. hyopneumoniae, M. hyorhinis, M. pulmonis, U. diversum, U. urealyticum*, and *U. parvum* [17,102,120–129]. Some of the characterized nucleases are listed in Table 2. Certain nucleases contain distinct functional domains, such as the TNASE_3 domain or glutamic acid-, lysine-, and serine-rich (EKS) regions. For example, both *M. gallisepticum* MGA_0676 and *M. bovis* MbovNase contain a TNASE_3 domain, which is essential for nuclease activity, cytotoxicity, apoptosis, and nuclear translocation [71,103,130,131]. In contrast, the EKS region of *M. pneumoniae* Mpn133 is essential for binding and internalization in A549 cells and nuclear localization of mycoplasma proteins within the host cells, but it is not involved in the enzymatic activity of Mpn133 [17,132]. Mycoplasma nucleases are not only considered to perform a metabolic function in the generation of nucleotide precursors from host or bacterial nucleic acids released by normal and death cell, but also serve as a significant immune evasion factor for certain mycoplasmas [133,134]. The nucleases-mediated invasiveness is predominantly reflected in the following aspects: (I) degradation of neutrophil extracellular traps (NETs), thereby promoting evasion of innate immunity (e.g. *M. agalactiae* MAG_5040, *M. hominis* MHO_0730, *M. pneumoniae* Mpn491, and *M. bovis* MnuA) [134–137]; (II) modulation of the expression of certain proinflammatory cytokines, such as, *M. hyopneumoniae* Mhp597 that upregulates the expression of inflammatory genes and downregulates the expression of type I IFN [125].

**Table 2.** Biological characteristics of certain nucleases in mycoplasmas

Species	Name	Divalent cation	Inhibitory agent	Subcellular location	Nuclease activity	Substrates	References
*M. pneumoniae*	Mpn133^a^	Ca^2+^	Mg^2+^,Mn^2+^, K^+^, Na^+^	Membrane-associated	Undefined	RNA, ssDNA, dsDNA	[132]
	Mpn491	Mg^2+^	Zn^2+^, EDTA	Secreted	DNase	DNA	[136]
*M. genitalium*	MG_186 ^a,b^	Ca^2+^	Zn^2+^, Mn^2+^, EGTA, EDTA	Membrane-associated	Endo-/Exo-	RNA, ssDNA	[133]
*M. hominis*	MHO_0730^b^	Ca^2+^	Mg^2+^, K^+^, Na^+^	Membrane-exposed	Endo-/Exo-	RNA. dsDNA, ssDNA	[135]
*M. penetrans*	P40	Ca^2+^, Mg^2+^	Zn^2+^, Mn^2+^, EGTA, EDTA	Membrane, intracellular	Endo-	RNA. dsDNA, ssDNA	[128,129]
*M. bovis*	MnuA	Ca^2+^, Mg^2+^	Undefined	Membrane-exposed	Endo-/Exo-	plasmid DNA	[121,137]
	MbovNase	Ca^2+^	Undefined	Secreted, membrane-bound	Endo-	RNA	[131]
*M. hyopneumoniae*	Mhp379 ^a,b^	Ca^2+^	Mg^2+^, K^+^, Na^+^	Membrane-exposed	Endo-/Exo-	RNA, ssDNA	[126]
	Mhp597^a^	Ca^2+^, Mg^2+^	Undefined	Secreted	Undefined	RNA, ssDNA, dsDNA	[125]
*M. hyorhinis*	Undefined	Ca^2+^, Mg^2+^	Zn^2+^, EGTA, EDTA	Undefined	Endo-	internucleosomal DNA	[127]
*M. gallisepticum*	MGA_0676	Ca^2+^	Mg^2+^, Cu^2+^	Membrane-exposed	Endo-/Exo-	plasmid DNA, ssDNA, dsDNA, RNA	[130]
*M. agalactiae*	MAG_5040^b,c^	Mg^2+^	Ca^2+^	Membrane-exposed	Endo-/Exo-	ssDNA, dsDNA	[134]
*M. meleagridis*	Mm19	Mg^2+^	Undefined	Membrane-exposed	Endo-/Exo-/RNase	ssDNA	[123]

a. thermostable nucleases

b. show sugar-nonspecific nucleases activity

c. nuclease activity can be enhanced by Na^+^ and K^+^

#### Immunoglobulin proteases

Certain mycoplasmas degrade immunoglobulins (Ig) as a strategy to subvert the host immune system. Based on the available data, mycoplasmas utilize three mechanisms for degrading Ig. The first mechanism is the secretion of IgA protease, as reported in *U. urealyticum* and *U. parvum*, which participates in facilitating microorganism colonization and host immune system evasion on the mucosal surface by proteolytic activity against IgA_1_ [15,138–140]. The second mechanism is retaining the Ig binding protein and Ig protease (MIB-MIP) system to degrade IgG. For instance, Mmc carries the MIB-MIP system that requires sequential assembly, in which MIB first captures IgG by binding to the Fv region of IgG, and then MIP binds to the MIB-IgG complex to exert serine protease activity, followed by cleavage of the domains between IgG V_H_ and C_H_3, thereby contributing to evasion of host immunity [7]. *M. mycoids subsp. mycoids* also expresses the MIB and MIP homologous proteins, whereas *M. hominis, U. urealyticum, U. parvum*, and *U. diversum* contain the MIB-MIP-encoding genes, but whether these genes are involved in the degradation of IgG remains unknown [103]. In addition, some mycoplasmas, such as *M. genitalium* and *M. pneumoniae*, lack the MIB-MIP system, but they can express protein M and DUF31-annotated genes in cells and play a role similar to that of the MIB-MIP system [7,141]. The third mechanism of degrading Ig is the expression of cysteine proteases (CysP). *M. synoviae* and *M. gallisepticum* CysP can cleave chicken IgG into Fab and Fc fragments, thereby facilitating their survival in the host [142].

#### Antioxidant enzymes

During infection, mycoplasma inevitably encounters oxidative stress owing to the host immune response. Certain mycoplasma can also express various antioxidant enzymes including methionine sulfoxide reductase (MsrA), organic hydroperoxide reductase (Ohr), osmotically inducible protein C (OsmC), superoxide dismutase (SOD), catalase, ClpB, thioredoxin reductase, thiol peroxidase, and peroxiredoxin [85,124,143–146]. These enzymes can effectively protect mycoplasmas from oxidative damage imposed by the host, and significantly increase their survival in the host. For instance, MsrA is an antioxidant enzyme localized primarily in the cytosol and is important for the maintenance of cytadherence and virulence potential in *M. genitalium, M. hyopneumoniae*, and *U. parvum* [102,145,147,148]. MsrA-deficient mycoplasmas have a reduced ability to interact with host cells (including adhesion and cytotoxicity), reduced viability in hamsters, and higher susceptibility to H_2_O_2_ and phagocytosis [102,147,148]. Das et al. had demonstrated that pretreatment with MsrA significantly abrogates *M. genitalium-*induced HeLa cell necrosis and TNF-α secretion [149], indicating that MsrA plays protective roles in the modulation of host cellular processes.

Ohr is also a type of anti-oxidative stress molecule identified in mycoplasmas in recent years, which belongs to the OsmC superfamily, and has been involved in resistance to oxidative stress damage. Ohr molecules exist in various mycoplasmas, such as *M. pneumoniae* (MPN668), *M. genitalium* (MG_454), and *M. gallisepticum* (MGA1142) [150–153]. MPN668, MGA1142, and MG_454 are novel Ohr proteins with hydroperoxidase activity on both inorganic and organic hydroperoxides, but MGA1142 preferentially degrades linear organic peroxide. MGA1142 transcription is upregulated under ethanol and downregulated under osmotic stress conditions, whereas MG_454 is strongly induced by osmotic shock and heat shock [151–153]. OsmC is also a structurally and biologically homologous OsmC superfamily protein. *M. genitalium* MG_427 is an OsmC-like protein that is endowed with hydroperoxide reductase activity, but its transcription is downregulated under osmotic shock and ethanol conditions [152]; therefore, its physiological function requires further study.

Theoretically, some glycerol-utilizing mycoplasmas, such as *M. iowae*, can produce H_2_O_2_ during glycerol metabolism, but no detectable H_2_O_2_ was produced under various experimental conditions; this may be interpreted as being due to the existence of a catalase. Indeed, Pritchard et al. successfully cloned the active catalase, katE, which can catalyze the degradation of H_2_O_2_ efficiently [154,155]. The results illustrate that catalases can protect the mycoplasmas from environmental H_2_O_2_ and are beneficial for its survival in the host. Similar catalases also exist in *M. arginini* [156]. Furthermore, there are studies speculating that SOD activity exists in some mycoplasmas but currently, no SOD coding genes are available in the fully sequenced mycoplasmas, although the SOD gene appears to be unique to *M. haemofelis* among the mycoplasmas [157]. These antioxidant stress molecules can antagonize oxidative stress damage to varying degrees, making it beneficial to circumvent the immune system.

#### Sialidases and hyaluronidases

Sialidase/neuraminidase is a pathogenic enzyme which can catalyze the hydrolysis of sialic acid and participate in the destruction of ECM, colonization, tissue invasion and apoptosis for microorganisms [158]. It has previously been thought that there had been a lack of corresponding enzymes in mycoplasmas, but evidence has proven otherwise. For example, several canine mycoplasmas, such as *M. canis, M. cynos*, and *M. molare*, show secreted sialidase activity, while strictly cell-associated sialidase activity is observed in *M. alligatoris, M. synoviae*, and *M. gallisepticum* [159–165]. Although destruction of the sialidase gene of *M. gallisepticum* resulted in the absent of sialidase activity and reduced tracheal lesions, the genetic complementation of sialidase activity could not be restored to wild-type virulence, proving that sialidase is not necessary for the virulence of *M. gallisepticum* [166]. Furthermore, although *M. neurolyticum, M. gallisepticum, M. synoviae, M. anseris, M. cloacale, M. pullorum, M. alligatoris, M. meleagridis*, and *M. corogypsi* show neuraminidase enzymatic activity, this activity varies among species or cultures [160,167–169]. The *M. synoviae* neuraminidase NanH can desialyze chicken tracheal mucus glycoproteins and chicken IgG heavy chain, thereby contributing to *M. synoviae* colonization and persistent infections [160,168].

Hyaluronidases can be found both in *M. alligatoris* and *M. crocodyli*, but sialidase is absent in *M. crocodyli*, which may explain why it is less virulent than *M. alligatoris* [161]. The sialidase and hyaluronidase of *M. alligatoris* act in rapid organism invasion, dissemination, necrosis, nutrient scavenging, and induction of pulmonary fibroblast apoptosis [161,163].

### Biofilms

Biofilms are bacterial communities attached to the surface of a biotic or inanimate object, usually surrounded by bacterial extracellular macromolecules, such as proteins, polysaccharides, DNA, RNA, lipids, and phospholipids [6,170,171]. Due to the physical barrier effect of biofilms and the special microenvironment within the membrane, biofilms contain the following peculiar features: (I) resistance to environmental stressors, antibiotics, antibodies, and host defense; (II) bacterial persistence in the environment and inside the host leading to chronic infection, or paroxysmal acute infection when planktonic cells are periodically released from the biofilm [170]; (III) continuous host or tissue damage since the attracted functionally frustrated phagocytes release phagocytic enzymes under these conditions [77].

To date, at least three human pathogenic mycoplasmas are known to form biofilms. The most common biofilm-forming mycoplasmas are *U. urealyticum* and *U. parvum*. A clinical survey indicated that 9 out of the 11 clinical isolates of *U. urealyticum* and *U. parvum* were found to form biofilms, but these 9 strains were completely sensitive to clarithromycin regardless of the type of growth [172]. *M. pneumoniae* can form a volcano-like biofilm; P1, neuraminidase, and sialyllactose are involved in adherence to the surface and biofilm formation [173]. Different strains manifest diverse potential for biofilm formation within *M. pneumoniae*; for example, the type 2 strain formed more prolific biofilms than the type 1 strain [174]. The biofilms formed by *M. pneumoniae* possess a characteristic tower structure, which endue the function of protecting them from the lytic effects of the complement system and gramicidin [175]. Moreover, as the biofilms mature, *M. pneumoniae* cells undergo morphologic changes; however, the altered motility has no more than a minor role in biofilm development [176]. Feng et al. also discovered that H_2_O_2_ and H_2_S production and CARDS TX levels peaked at the early stage of biofilm formation and decreased over time, while antibiotic and complement resistance increased over time [177], suggesting that the virulence of individual bacteria may be reduced after *M. pneumoniae* reaches a chronic infection stage. Although some reports have shown that *M. hominis* is detected in the “amniotic fluid sludge” regarded as a biofilm, it remains controversial whether *M. hominis* can form biofilms since it was mixed with other bacteria in those experiments [178].

The biofilm-forming mycoplasmas in animals include *M. pulmonis, M. putrefaciens, M. cottewii, M. yeatsii, M. agalactiae, M. bovis, M. dispar, M. arginini, M. gallisepticum, M. suis*, and *M. hyopneumoniae* [6,71,77,80,112,113,156,170,171,179–182]. *M. pulmonis* expresses variable surface antigens (Vsa). The biofilm form of *M. pulmonis* is Vsa isotype independent, but is associated with the length of Vsa; short Vsa with 0–5 tandem repeats forms a biofilm attached to polystyrene and glass, whereas, the long Vsa with many repeats forms micro-colonies that float spontaneously in the medium [6,112,113], indicating that long Vsa proteins sterically impede connections between the mycoplasma cell surface and the environment. In addition, Simmons et al. have observed the formation of biofilms by *M. pulmonis* in tracheal organ cultures and experimentally infected mice, which show similar functions and tower structures as formed *in vitro* [183].

Experiments have confirmed that in a glass coverslip model with an air-liquid interface, *M. putrefaciens, M. cottewii, M. yeatsii, M. agalactiae*, and *M. bovis* produce prolific biofilms, all of which are highly resistant to stressors, such as heat and desiccation [77,80]. Conversely, Mmm small colonies (SCs) cannot produce biofilms under this condition [170]; however, when attached to a solid surface, Mmm SCs produce biofilms and are resistant to heat, osmotic shock, and oxidative stress [144]. Interestingly, several glycolytic enzymes or extracellular matrix-binding adhesins, such as pyruvate dehydrogenase and EF-Tu, are upregulated when Mmm SC is grown as an adherent biofilm [144], which demonstrates that surface adherence may be an essential process, and that glycolytic enzymes may play a role in biofilm formation and disease initiation by Mmm SC. Correspondingly, the production of biofilm by *M. putrefaciens* and *M. bovis* did not affect the minimal inhibitory concentrations of any antibiotic tested [71,77,170]. Additionally, it is known that all *M. bovis* isolates can produce biofilms and are correlated with different molecular types or Vsp species. Among them, the expression of VspB and VspO, but not VspF, was found in all biofilm-producing isolates. Nevertheless, the ability of *M. bovis* to produce biofilms does not necessarily correlate with its pathogenicity [77]. Furthermore, *M. arginini* camel isolates also form biofilms on the surface of polystyrene, but their biofilm-forming ability is rather weak owing to the poor production of catalase [156].

Nevertheless, a considerable proportion of *M. gallisepticum* strains can produce biofilms, the most potent of which is the Nobilis MG 6/85 strain [181]. Intriguingly, the Nobilis MG 6/85 is an attenuated vaccine strain and its biofilm formation is not necessarily associated with pathogenicity. Otherwise, all *M. gallisepticum* strains from house finches produce biofilms, but no difference was observed in the biofilm density between these strains [165], which implies that the formation of biofilms was independent of the house finch conjunctivitis caused by *M. gallisepticum*.

*M. suis*, formerly known as *Eperythrozoon* spp. of the family Anaplasmataceae in the order Rickettsiales, was reclassified as a distinct new cluster in the genus mycoplasma based on 16S rRNA gene sequences [184]. Although there is a close phylogenetic relationship between *M. suis* and organisms belonging to the pneumoniae group of mycoplasmas, this zoonotic pathogen colonizes erythrocytes, and takes the latter as the preferred target, causing infectious anemia in pigs [185]. Recent evidence shows that *M. suis* can also interact with the porcine endothelial cells (ECs) and shaping biofilm-like micro-colonies through a budding-like replication mechanism, which makes them capable of propagating on ECs and thereby protect the organism from antibiotics and immune factors to establish persistence infections [182]. *M. hyopneumoniae* can produce biofilms both *in vivo* (swine respiratory tract) and *in vitro* (PK-15 monolayers and abiotic surface) [180]. In addition, its biofilm contains extracellular DNA (eDNA), which initiates the process of biofilm formation [171].

In conclusion, most mycoplasma biofilm production discussed above was observed *in vitro*, which may not mimic the physiological condition during the infection; therefore, further experiments are needed to investigate the role of biofilms in the pathogenicity of mycoplasmas.

## METABOLITES

### Hydrogen peroxide

The description of hemolysis of erythrocytes was originally discovered when the *Pleuropneumonia bovum* strains were growing in the presence of blood [186]. Subsequently, the catalase and horseradish peroxidase experiments confirmed that the mechanism of hemolysis was caused by mycoplasma-produced H_2_O_2_ or organic peroxide. Indeed, many mycoplasmas, such as *M. pneumoniae, M. homins, M. bovis, M. agalactiae*, Mmm SC, *M. dispar, M. bovirhinis*, Mccp, *M. gallisepticum, M. canis*, and *M. hyopneumoniae* can metabolize glycerol as carbon source to synthesize ATP, and ultimately produce H_2_O_2_ as virulence determinants [3,9,17,80,85,165,179,187–196]. The glycerol metabolism mechanism of mycoplasma is becoming clearer. Generally, when glucose is present as the preferred carbon source, the expression of glycerol metabolism-related genes is suppressed [9,197]. Only when glycerol or its derivatives, such as glycerophosphocholine (GPC), is available, glycerol metabolism-related genes are expressed. Glycerol is usually absorbed by the glycerol import system GtsABC or the facilitator factor GlpF, and then phosphorylated by glycerol kinase (GlpK) to glycerol 3-phosphate (G3P). GPC is taken up by glycerophosphocholine importer (GlpU) [179], and then degraded by glycerophophodiesterases (GlpQ) to G3P [9,17]. Next, G3P is oxidized by L-α-glycerophosphate oxidase (GlpO or GlpD) and H_2_O_2_ is released as a byproduct [179]. H_2_O_2_ plays a role in lipid peroxidation, cell damage and death, erythrocyte lysis, and ciliary action inhibition [77].

*M. pneumoniae* is the most thoroughly studied human pathogenic mycoplasma concerning glycerol metabolism. *M. pneumoniae* generates H_2_O_2_ during the metabolism of glycerol by GlpD and GlpQ [9,17,198]. The formation of H_2_O_2_ is essential to the cytotoxicity of *M. pneumoniae* and can cause host cell damage, peroxidation of lipids, and oxidation of heme, rather than the lysis of erythrocytes [8,9,17]. Moreover, *M. homins* can also produce H_2_O_2_ via GlpO [191].

Hydrogen peroxide production was identified in all types of *M. bovis* and the wild strains of *M. agalactiae*, although the H_2_O_2_-producing ability was different among these isolates [77,78,80,192]. *In vitro* serial passage of a high H_2_O_2_-producing strain (119B96) of *M. bovis* led to reduced H_2_O_2_ levels, but did not affect substrate oxidation compared with that of the parent strain. After the 50 ^th^ passage, H_2_O_2_ was reduced to approximately 50%, which may be linked to the loss of a 32-kDa protein, as indicated using sodium dodecyl sulfate-polyacrylamide gel electrophoresis [77,192]. Comparative genomics demonstrated that certain genes with single-nucleotide polymorphisms and indels in *M. bovis*, but outside the 14.2-kb deleted region, might be associated with the altered H_2_O_2_ production [195]. Zhu et al. indicated that eDNA is required for *M. bovis* H_2_O_2_ generation [194].

Under physiological concentrations of glycerol, the highly virulent strains of Mmm SC carry a *gtsABC* transport system that can produce high levels of H_2_O_2_ and exhibit strong hemolytic activity, whereas low virulent strains lacking the *gtsB* and *gtsC* genes are contrary to the high-virulence strains [189]. In Mmm SC, the glycerol in the interstitial fluid was actively taken up via the GtsABC or the less efficient GlpF, and subsequently phosphorylated to G3P by GlpK. Finally, the G3P is oxidized to dihydroxyacetone-phosphate to enter in the glycolytic pathway with the release of H_2_O_2_. This toxic metabolite and the accompanying ROS can enter the host cell by intimate contact of Mmm SC and the cell membrane, and cause cell injury and inflammatory signaling cascade [190].

*M. dispar, M. bovirhinis*, and Mccp can also produce H_2_O_2_ in the presence of glycerol [3,85,179]. The genomes of *M. dispar* and *M. bovirhinis* lack the *gtsABC* gene cluster and *glpO*, but they do contain a *glpF-glpK-glpD* gene cluster [3,179]. In contrast, both *gtsABC* and *glpF-glpK-glpD* gene clusters are present in the Mccp genome [85], and are relevant to virulent. Furthermore, *M. gallisepticum* and *M. canis* can also metabolize glycerol and form H_2_O_2_ [187,193], but not *M. hyopneumoniae*, as only certain pathogenic strains are capable of generating H_2_O_2_ [191].

Interestingly, the generation of H_2_O_2_ is not necessarily linked to the severity of pathology for certain strains. For example, H_2_O_2_ formation by *M. bovis* do not differ between the isolates from caseonecrotic bronchopneumonia and non-inflamed lungs or other forms of pneumonia [71,199]. In addition, although the GlpF, GlpK, and GlpO mutants of *M. gallisepticum* were incapable of producing H_2_O_2_, these mutants remained fully virulent in the chicken respiratory tract [165,193]. Likeweise, the naturally attenuated strains retain the capacity to produce H_2_O_2_ in cultures [193]. Furthermore, although Mmm SC type strain PG1 has been shown to release a large amount of H_2_O_2_ at physiological concentrations of glycerol, the cytotoxicity observed was almost negligible; exogenous addition of H_2_O_2_ to the axenic medium is not sufficient to cause cytotoxicity [188]. Therefore, these results demonstrate that H_2_O_2_ may have a limited function in the pathogenesis of certain mycoplasmas.

### Ammonia

Urease is the first metabolic enzyme found in ureaplasmas. Some ureaplasmas, such as *U. urealyticum, U. parvum*, and *U. diversum* express urease, which can hydrolyze urea to produce ATP and simultaneously produce CO_2_ and NH_3_ [2,102,103,138–140]. Ureaplasmas lack the ability to assimilate NH_3_ to glutamine and glutamate, leading to increased pH in the surrounding environment, thereby exerting toxic effects and tissue injury [139,140]. Other potential pathogenic effects of NH_3_ production including fatal hyperammonemia syndrome and the formation and precipitation of struvite in the urinary tract [200,201]. A study also showed that the urease activity of *U. urealyticum* was higher than that of *U. parvum* [15], suggesting that *U. urealyticum* may trigger more severe localized pathological damage.

Arginine hydrolysis is another source of NH_3_. Most of the non-fermentative mollicutes and some fermentative species possess the arginine dihydrolase pathway to generate energy, which results in the production of ornithine, ATP, CO_2_, and NH_3_ [2]. A certain concentration of NH_3_ can be produced by a number of arginine-utilizing mycoplasmas, such as *M. hominis, M. gallinarum, M. fermentans, M. pirum, M. salivarium, M. iowae*, and *M. meleagridis* [2,102,202,203]. Owing to the consumption of arginine, arginine-utilizing mycoplasmas are toxic to cultured mammalian cells and may induce immunosuppression [202,204].

### Hydrogen sulfide

The hemolysis of *M. pneumoniae* was thought to be attributed to the H_2_O_2_ produced during glycerol metabolism [205]. However, Großhennig et al. had found that the erythrocytes under a low concentration environment remained intact but had unexpected α-hemolytic activity in the supernatant following *M. pneumoniae* infection [8]. After knocking out GlpO, *M. pneumoniae*’s ability to metabolize glycerol to produce H_2_O_2_ is reduced, so the mutant lost the ability to cause α-hemolysis, which impelled them identified an unusual cysteine desulfurase/desulfhydrase HapE, which exhibits cysteine desulfurase and cysteine desulfhydrase double activities, and can convert cysteine to pyruvate and H_2_S to cause the modification of hemoglobin and damage to the erythrocytes [8,17]. In other mycoplasmas such as *M. genitalium, M. gallisepticum, M. amphoriforme*, and *U. parvum*, HapE homologous sequences also annotated in their genomes, of which, the highest homology is with *M. genitalium* MG336, but its function remains unclear. H_2_S is a novel potential virulence factor for *M. pneumoniae*, which can cause the modification of heme and lysis of erythrocytes to allow the bacteria to get efficient access to the host’s resources together with other virulence determinants such as H_2_O_2_ [8,17].

## EXOTOXINS

### Community-acquired respiratory distress syndrome toxin

The discovery of CARDS TX of *M. pneumoniae* challenged the previously held belief that mycoplasmas are devoid of exotoxins. CARDS TX was originally a membrane-associated, surfactant protein A (SP-A)-binding, ADP-ribosylating, and vacuolating toxin identified by Kannan and Baseman when studying the pathogenic factors that may be responsible for respiratory epithelial cell damage by *M. pneumoniae* [11]. It is encoded by the *mpn372* gene and is a protein of approximately 68-kDa, containing 591 amino acids. Among them, amino acids 1–239 are 27% homologous with pertussis toxin catalytic subunit S1 [11,20]. The N-terminus of CARDS TX has ADP-ribosyltransferase (ART) activity, and its C-terminus is essential for vacuolization [206,207]. Three-dimensional structural analysis has revealed that CARDS TX is a triangular molecule with three domains (D1–D3) [208]. D1 possesses ART activity, whereas D2 and D3 are involved in receptor recognition, cell surface binding, internalization, and vacuolization [209]. Furthermore, Balasubramanian et al. had demonstrated that a disulfide bond is formed between cysteine C230 and C247 of CARDS TX, which is essential for ADP-ribosylating and vacuolating activities, for maintaining the conformational stability of the toxin, and for appropriately performing cytopathic effects, but does not affect the binding, internalization, and retrograde transport properties [210].

In patients diagnosed with *M. pneumoniae* pneumonia, a significant seroconversion of CARDS TX has been observed [11], indicating that the toxin is synthesized *in vivo* and possesses highly immunogenic epitopes. When *M. pneumoniae* was co-cultured with host cells, CARDS TX expression was significantly increased compared with that in *in vitro* inanimate medium, thereby supporting the fact that the synthesis of this toxin depends on the interaction between host cells and *M. pneumoniae* [17]. At the same time, *M. pneumoniae*-infected mice showed significantly increased amount of CARDS TX protein in lung tissues compared with that cultured in SP-4 liquid medium [211]. This suggested that the transcription and translation processes of CARDS TX are strengthened after *M. pneumoniae* receives the environmental stimulation signal from the host cell. In addition, different strains appear to exhibit multifarious CARDS TX producing capabilities. Techasaensiri et al. suggested that although the S1 strain (subtype 2) and two M129 strains (M129-B7 and M129-B9, subtype 1) can produce CARDS TX, the former expresses more CARDS TX in contrast to the latter two [212]. The differential expression of CARDS TX in these subtype strains was further verified using transcriptome and proteome analysis [213]. This is consistent with a previous report that subtype 2 strains are more prolific in biofilms [174], which indicates that subtype 2 is more virulent.

CARDS TX selectively binds phosphatidylcholine and sphingomyelin over other membrane lipids [208]. Nevertheless, CARDS TX is known to bind to SP-A, which may contribute to additional colonization and pathogenic pathways [11,211]. Krishnan et al. showed that CARDS TX can bind to certain cell types in a dose- and time-dependent manner, followed by rapid internalization by clathrin-associated endocytosis via a temperature-sensitive mechanism [17,214]. Interestingly, CARDS TX internalization can also occur in SP-A-deficient cells [214], demonstrating the presence of alternative binding receptors. Sudha et al. discovered that CARDS TX, especially the C-terminus of CARDS TX, binds recombinant AnxA2 in a specific, dose-dependent manner [215]. Cells pretreated with anti-AnxA2 monoclonal antibody or AnxA2 small interfering RNA (siRNA) show reduced binding, internalization, and vacuolization, while this is not the case in AnxA2 siRNA-transfected HepG2 cells [215]. These results suggest that AnxA2 may serve as another functional receptor for CARDS TX in certain cells and participate in toxin-related pathogenic events.

CARDS TX can activate NLRP3 inflammasome by catalyzing the ADP-ribosylation of NLRP3, thereby cleaving pro-IL-1β to mature IL-1β [17,20,207]. CARDS TX mutants lacking the ART activity fail in binding and internalization, and thereby are unable to activate the NLPR3 inflammasome [216], indicating that ADP-ribosylation is a unique post-translational modification that is essential for the activation of NLRP3 inflammasome. In short, once the CARDS TX is trafficked to early and late endosomes, it undergoes retrograde transport from the Golgi complex to the endoplasmic reticulum through an unusual KELED motif located at the junction of D1 and D2 domains, causing ADP-ribosylation-dependent NLPR3-mediated IL-1β release and induces the formation of vacuoles, which subsequently exert cytotoxicity [209].

The CARDS TX, both produced by natural infection and the recombinant form (rCARDS TX), can elicit profound inflammatory responses [217,218]. In a murine model of *M. pneumoniae* infection, the level of CARDS TX in bronchiolar lavage fluid was positively correlated with pathological changes in the lungs and the severity of the disease [218]. Moreover, a clinical study assessing children with refractory *M. pneumoniae* pneumonia demonstrated that CARDS TX and TNF-α co-expression is associated with enhanced pulmonary inflammatory infiltration and mucus secretion [219]. The exposure of rCARDS TX to the pulmonary compartment in both mice and baboons causes a prolonged inflammatory response, infiltration of inflammatory cells around the bronchus and blood vessels, and airway dysfunction. Such inflammation is characterized by rapid expression of proinflammatory cytokines and chemokines with the concurrent development of lymphocytic inflammation [220]. In addition, intratracheal instillation of rCARDS TX is sufficient to elicit allergic-type pulmonary inflammation or to exacerbates ovalbumin-induced asthma-like inflammation in mice, and is characterized by eosinophilia, mucus metaplasia, airway hyperreactivity, and T-helper-2 type cytokine and chemokine expression [217,221]. The inflammatory pathology and lung dysfunction are largely dependent on CD4^+^ T cells. However, Maselli et al. confirmed that the kinetics of the T cell response of primates to CARDS TX or *M. pneumoniae* is delayed than that of murines [222]. Both single- and double-dose rCARDS TX are equally sufficient to increase the total IgE and rCARDS TX-specific IgE levels in serum [223]. The latter can bind to the N-terminal peptide of CARDS TX and cause mast cell degranulation. The N-terminal peptide of CARDS TX can also cause mast cell degranulation [223], indicating that CARDS TX generates functional lgE and that the mast cell-lgE axis is critical for CARDS TX-mediated allergic inflammation. Furthermore, CARDS TX not only causes inflammation of the respiratory system but can also mediate the deterioration of ciliary movement of the respiratory tract, epithelial cell damage, and apoptosis [11].

Recombinant CARDS TX also shows cytopathic effects in mammalian cells in tissue and organ cultures, eventually leading to cell death, which is consistent with the process during *M. pneumoniae* infection [220]. Vacuolization of the bronchial epithelium is another function of rCARDS TX observed in rCARDS TX-treated mice. Neutral red-uptake tests revealed that the rCARDS TX-induced vacuoles are acidic. The vacuoles originate from Rab9-associated compartments and late endosomes since they are rich in Rab9, LAMP1, and LAMP2; the ATPase inhibitor bafilomycin A1 and monensin can abrogate this vacuolization [17,224], demonstrating that vacuolization mediated by rCARDS TX is dependent on V-ATPase enzymes, which are also found in endosomes and lysosomes.

Although CARDS TX seems to be unique to *M. pneumoniae*, some homologous genes are present in *M. neurolyicum* and *M. iowae* (NCTC10166_00515 and P271_571, respectively). Comparative genome sequencing revealed that they shared 45.9% and 26.6% homology with CARDS TX, respectively. But their contribution to the virulence of these species remains to be investigated. Likewise, an ADP-ribosyltransferase also exists in *M. penetrans* (MYPE9110) [225], but its function remains unclear. Furthermore, although *M. genitalium* shares the highest homology with *M. pneumoniae*, no similar genes have been identified.

### Hemolysin

Except for the above-mentioned hemolytic activity by H_2_O_2_, a few mycoplasmas can also secrete hemolysin, which is thought to lyse erythrocytes by forming pores on the cell membrane to obtain membrane precursors [103]. In this way, mycoplasmas can efficiently acquire macromolecular precursors from host and simultaneously exert an important pathogenic role which contributes to the survival of mycoplasmas.

Certain human pathogenic mycoplasmas, such as *U. parvum, U. urealyticum*, and *M. penetrans*, display obvious hemolysin activity *in vitro. U. parvum* serovar 3 and *U. urealyticum* express hemolysin A (hlyA), and hlyA and hlyC, respectively [103]. Of these, *U. parvum* hlyA exhibits hemolytic and cytotoxic activities, whereas *U. urealyticum* hlyA has a bearing on hemolysis and hlyC is involved in H_2_O_2_ production [226]. Furthermore, various *M. penetrans* strains show hemolytic activity following incubation with sheep, horse, chicken, and human erythrocytes, although this activity has not been previously found in mouse erythrocytes; the spent culture supernatant of *M. penetrans* also has hemolytic activity that depends on the reducing agent cysteine [10]. These results indicate that *M. penetrans*-derived hemolysin can bind to specific receptors on the erythrocytes, and cysteine residues are required for hemolytic activity.

Hemolysin also exists in animal mycoplasmas. *M. pulmonis* contains a bovine serum albumin-dependent, membrane-associated hemolysin, which recognizes cholesterol in the opposing membrane once attached, causing erythrocyte lysis [227]. Chambaud et al. had found that the *M. pulmonis* hemolysin gene *MYPU_1701* is homologous to certain genes in *U. urealyticum*, but no homolog was found in *M. pneumoniae* and *M. genitalium* [124]. Similarly, *U. diversum* encodes a putative hemolysin protein gudiv_91, which is 63.1% identical to the hemolysin found in human ureaplasmas [103]. Moreover, a similar membrane-associated hlyA gene (*MCAP_0055*) has been reported in *M. capricolum*, which may account for the hemolytic activity of the spent culture medium of *M. capricolum* [228].

Recently, an hlyC-related sequence has been found in the genome of *M. hyorhinis* strain HUB-1. After mutation by the oriC-plasmids, the hemolytic ability of the culture supernatant on mouse erythrocytes was shown to be significantly decreased. This is the first time that a particular gene was associated with a hemolytic phenotype [229]; however, further investigations are warranted to verify the pathogenic role of *M. hyorhinis*. Nevertheless, there are three hemolysin genes (*hlyA, hlyB*, and *hlyC*) present in the genomes of *M. conjunctivae* and *M. hyopneumoniae* [230]. Although these hemolysins are not as important as those in other pathogenic mycoplasmas, it cannot be ruled out that these hemolysins contribute to the pathogenicity of these mycoplasmas. Hemolytic activity has also been found in some *M. arginini* camel isolates [156], but the mechanism is not yet understood.

Although hemolysin-like genes or proteins do exist in certain mycoplasmas, such as the *M. pneumoniae* hemolysin-like gene (*VXpSPT7_orf424*) [231], *M. bovis* hemolysin-related protein (MMB_0258) [143], Mccp hemolysin A gene (*MCCG_0074* and *XDU01000067*) [85,232] and the newly discovered “Candidatus *M. girerdii*” hemolysin homologous protein [233], the exact role of these hypothetical hemolysins remains to be established. Similarly, all glucose/arginine-utilizing mycoplasma species, except for *M. hominis, M. penetran*s, *M. arthritidis, M. hyosynoviae, M. pulmonis* PG34, *M. fermentans* spp. *incognitus*, and *M. meleagridis*, contain significant membrane-associated hemolysin activities [234]. Therefore, hemolysin appears to belong to a unique class of bacterial toxins, and its pathogenesis warrants further exploration.

## PATHOGENIC ENZYMES

### Lipolytic enzymes

Several mycoplasmas express multiple lipolytic enzymes, including lipase, phospholipase, and other forms, which are necessary for mycoplasma metabolism, and are also involved in a variety of pathogenic processes. *M. hyopneumoniae* P65 surface lipoprotein is a lipolytic enzyme with lipase and esterase activities that can hydrolyze short-chain fatty acids on the host cell membrane as its own energy source, but its pathogenic role remains unknown [235]. In addition, *M. bovis* surface-localized heparin-binding hypothetical adhesin MilA is an immunoreactive, multifunctional enzyme that comprises lipase, ATPase, lipid-binding, and glycosaminoglycan-binding activities, thus contributing to the pathogenesis of *M. bovis* infection [12]. *milA* homologues have been found in the genomes of numerous mycoplasmas, including *M. agalactiae, M. fermentans, M. hyopneumoniae, M. arginini*, and *M. gallisepticum*. The presence of the Lipase_GDSL_2 motif is a common characteristic of these *milA* homologous genes [12]. Similarly, comparative genome sequencing manifested that some lipase homologous genes have also been found in *M. pneumoniae* and *M. genitalium*, but their functions in pathogenesis remain to be fully understood.

Phospholipase, another metabolic enzyme of mycoplasmas, primarily plays a pathogenic function by destroying the integrity of the host cell membrane or by facilitating mycoplasma invasion. Phospholipase activity is identified in multiple mycoplasmas including *M. fermentans* (phospholipase C, PLC), *M. penetrans* (PLC and PLA_1_), *M. hyorhinis* (PLA and glycerophosphodiesterase), *U. diversum* (PLC), *U. urealyticum* (PLA_1_, PLA_2_ and PLC), and *U. parvum* (PLA_1_, PLA_2_ and PLC) [138,140,198,236]. Of these, *M. penetrans* PLA_1_ may trigger specific signal cascades in the host cell, thereby affecting HIV replication [236]. *M. hyorhinis* PLA and glycerophosphodiesterase participate in invading host eukaryotic cells, and PLA is also involved in the perturbation of the host membrane [198]. Interestingly, although PLD activity is not found in *M. hyorhinis*, two PLD conserved motifs exit in the cardiolipin synthase, which may be associated with the antagonistic effect of erythrocytes against *Staphylococcus aureus* β-hemolytic activity [237]. *U. urealyticum* and *U. parvum* exhibit PLA_1_, PLA_2_, and PLC activities; however, the corresponding coding genes have not been identified, and only a few PLD domain-containing proteins have been found in this bacterium; hence, their function needs further clarification. Similarly, *U. diversum* PLC also contributes to cell invasion [103,138]. Additionally, like *U. urealyticum*, several mycoplasmas, including *M. pulmonis*, have been reported to present PLC activity, yet it remains controversial whether this activity may ascribe to serum protein contamination rather than the PLC itself [234].

### Protease/peptidases

*M. capricolum subsp. capripneumoniae* strain 87,001 has been identified to possess an S41 peptidase that plays a role in stress response, metabolism, heat resistance, metal ion transport, and virulence [196,232]. A similar peptidase has also been found in Mmc (*MMCAP2_0241* gene), which confers this bacterium a proteolytic phenotype; disruption of this gene leads to an altered proteome profile, enhanced H_2_O_2_ production, increased sensitivity to heat shock, and decreased lactate dehydrogenase activity [196]. Moreover, *M. gallisepticum* has two putative peptidase coding genes: *MGA_1102* with a zinc peptidase-like motif and *MGA_1079* with a trypsin-like peptidase motif; these are essential for pathogenicity but not for colonization and persistent infection [13]. *M. hyopneumoniae* encodes three putative peptidases, type I signal peptidase (MhSpase I), Xaa-pro aminopeptidase (PepP), and oligoendopeptidase F (PepF). MhSpase I exerts a pro-apoptotic effect on PK15 swine epithelial cells by activating caspase-3, and this effect depends, at least in part, on the native conformation of the protein. PepP cleaves the N-terminal penultimate proline from bradykinin, substance P, and neuropeptide Y, while PepF cleaves full-length bradykinin, substance P, and neurokinin A. Both PepP and PepF modulate mucociliary clearance and inflammatory response [238–240]. Furthermore, the *M. bovis* Hubei-1 strain also secretes thirteen cytoplasmic peptidases and an extracellular cysteine protease (MMB_0708) that can degrade the host extracellular matrix proteins into oligopeptides to satisfy the *M. bovis* nitrogen requirement [143]. Similarly, *M. hominis* also presents a peptidase [102], but its function warrants further investigation.

### Phosphatases

Serine/threonine phosphatases (STPs) have been described in several bacterial pathogens as essential enzymes involved in phosphorylation-dependent signal transduction pathways and are often associated with the virulence of these organisms [241]. A typical STP is encoded by the *M. genitalium MG*_*207*, which can catalyze the dephosphorylation of threonine phosphate. The MG_207 mutant strain shows differential phosphorylated proteins and adherence phenotypes and reduced cytotoxicity [241], demonstrating that STP is critical for *M. genitalium* virulence. Furthermore, a eukaryotic-type STP of *M. pneumoniae* (PrpC) has been confirmed in gliding motility [242]. *M. synoviae* also encodes a PrpC [243], but its pathogenic role remains unknown.

### Ecto-ATPases

*M. hominis* surface-localized lipoprotein OppA, the substrate-binding domain of the oligopeptide permease, has been characterized as an ecto-ATPase [102,244]. OppA induces ATP-release from HeLa cells; subsequently, the discharged ATP is hydrolyzed by its intrinsic ATPase activity that ultimately results in apoptosis of the host cells [245]. Moreover, the cytadhesion of *M. hominis* also depends on the ecto-ATPase activity of OppA [246]. These results demonstrate that OppA affects not only nutrition uptake but also the host-pathogen interactions of *M. hominis*. Two OppA-coding genes were also identified in the genome of Mccp strain 87,001, but their functions remain unidentified [232]. *M. gallisepticum* possesses the *MGA_0220* gene that encodes a putative ATP-binding protein (OppD), which is involved in amino acid, dipeptide, and oligopeptide transport. OppD is required for pathogenesis as the mutant strain has been shown to have a reduced capacity to induce respiratory tract lesions and persist in the respiratory tract, but its ATPase activity remains to be investigated [13,247,248].

### Cytotoxic nucleases and nucleotidases

Mycoplasma nucleases are not only involved in the aforementioned degradation of NETs to escape the killing of neutrophils, but also have a direct pathogenic effect on host cells. For example, *M. pneumoniae* Mpn133 can induce apoptosis-like death of A549 mammalian cells through caspase-independent mechanism [132]; *M. genitalium* MG_186 can cause chromatin condensation, DNA degradation and apoptotic morphological changes [133]; similar cytotoxic nucleases can also be found in *M. penetrans* (P40), *M. hyopneumoniae* (Mhp379 and Mhp597) and *M. hyorhinis*, which cause host cell damage through multiple mechanisms [125–127,129]. Moreover, the *M. bovis* strain Hubei-1 secretes a 5ʹ-nucleotidase (MMB_0636) that can utilize host nucleotides as a substrate (such as ATP or ADP), which enhances macrophage death and damages the host tissue [78]. The gene encoding 5ʹ-nucleotidase has also been identified in the genome of *M. bovirhinis* [3].

### Glycosyltransferase and β-glucosidase

*M. pneumoniae* glycosyltransferase is involved in the biosynthesis of galactocerebroside, that is associated with Guillain-Barré syndrome [249]. In certain mycoplasmas and ureaplasmas, glycosyltransferase is involved in the formation of capsule, and thereby participating in anti-phagocytosis and promotes survival in the host [103]. Bgl is a 6-phospho-β-glucosidase that is related to the phosphoenolpyruvate-dependent sugar: Phosphotransferase system (PEP-PTS). Both of them participate in the incorporation and phosphorylation of β-D-glucoside [250]. Moreover, Mmm SC possesses a β-glucosidase encoded by *bgl*, while the gene diversity is associated with the degree of virulence of Mmm SC [251]. Comparative genome sequencing analysis of *bgl* revealed that the homology is also present in Mmc and *M. leachii*, but their function remains to be identified.

### Dihydrolipoamide dehydrogenase and Lipoate protein ligase

Dihydrolipoamide dehydrogenase, an E3 member of the PDH complex, is a virulence factor since a mutant of dihydrolipoamide dehydrogenase has demonstrated significant attenuation of *M. gallisepticum* virulence *in vivo* [252]. The dihydrolipoamide dehydrogenase gene has also been identified in *M. genitalium, M. bovis, M. capricolum*, and Mccp [78,232,253,254].

Lipoate protein ligase A (lplA) plays a role in the ligation of lipoic acid from host cells to the E2 subunit of the PDH complex to generate E2-lipoamide, which ultimately plays a crucial role in pyruvate metabolism [255]. lplA has been shown to be upregulated in the pathogenic 168 strain of *M. hyopneumoniae* [256]. Similarly, the *lplA* gene has also been identified in the genomes of *M. bovis* [78] and Mccp [85]. Nevertheless, further research on the pathogenic function of lplA in mycoplasmas is warranted.

## LIPIDS

Mycoplasmas can recruit and metabolize host lipids that are critical for their growth and survival. A detailed analysis of mycoplasma cell membrane lipids revealed that 35%–50% are neutral lipids, primarily unesterified cholesterol incorporated from the growth medium, and 50%–65% are polar lipids [257]. Owing to the unique biological characteristics of lipid molecules, the pathogenic role of lipids is largely unknown. At present, only a few lipids derived from mycoplasmas, such as *M. hominis, M. penetrans, M. pneumoniae, M. fermentans, M. orale*, and *M. gallisepticum*, have been studied [257], with *M. fermentans* being the most studied. The polar lipid fraction of *M. fermentans* is mainly composed of phosphatidylglycerol (PtdGro), phosphocholine-containing glycoglycerolipids (MfGL), glycoglycerophospholipids (GGPLs), ether lipids (MfEL) and its lyso form (lyso-MfEL) [1,257–259]. These versatile lipids participate in various pathogenic roles in adhesion, fusion, and cytokine secretion, thereby acting as important mediators of histopathology during mycoplasma infection.

There are two forms of MfGL, namely MfGL-I and MfGL-II, in which the latter being the primarily type. In the PG18 strain, MfGL-II enhances the fusion with Molt-3 lymphocytes in a dose-dependent manner. However, the MfGL-I-holding strains, M39 and M52, without MfGL-II, manifest the same fusion efficacy as Molt-3 cells and PG18 [1,260], suggesting that MfGL-I and MfGL-II may serve as fusogens. Nonetheless, the role of MfGL-II in cell membrane fusion remains controversial. For example, Rottem reported that MfGL-II is a bilayer stabilizing lipid and cannot undergo phase transition from a lamellar to an inverted configuration [1,261]. Similarly, using biophysical analysis, Gil et al. discovered that MfGL-II lacks fusogenic properties [261]. Therefore, the exact role of MfGL-II needs to be studied further. In addition to the fusion activity, MfGL-II also functions in moonlighting biological activities, including (I) triggering the inflammatory response of human monocytes and rat astrocytes by the activation of protein kinase C, secretion of nitric oxide (NO), prostagalandine E_2_, and TNF-ɑ, although much weaker than LPS; (II) yielding a strong immune response; and (III) increasing glucose utilization and lactate formation [262]. Nevertheless, the deacylation of MfGL-II or monoclonal anti-phosphocholine antibodies significantly reduces cytokine-stimulating activity, and the MfGL-II antiserum inhibits the attachment of *M. fermentans* to host cells, demonstrating that MfGL-II, especially the phosphocholine residue, plays a pivotal role in *M. fermentans*-host interaction [1,257,259–261,263].

There are two types of GGPLs, GGPL-I and GGPL-III, which are structurally similar to MfGL-II [257,259]. The monoclonal antibody of GGPL-III does not cross-react with GGPL-I, indicating that GGPL-III is a membrane-specific antigen of *M. fermentans*. GGPL-III alone displays a weak proinflammatory effect; however, it can promote both collagen-induced arthritis and nickel (Ni) allergy, although the effect is not as strong as that with LPS. The enhancement effect of GGPL-III on Ni allergy exists in mice deficient in either T cells or TLR4, but is significantly weaker in mice deficient in macrophages, IL-1, or the histidine decarboxylase [264], demonstrating that GGPL-III may be involved in certain types of chronic diseases, such as arthritis and allergy, through the innate immune pathway.

MfEL and lyso-MfEL are structurally similar to the platelet-activating factor (PAF) [258]. Although MfEL and lyso-MfEL do not show any PAF-like activity, they can demonstrate PAF-like activity including rapid bronchoconstriction, vasoconstriction, decreased tidal volume, and edema formation in isolated perfused and ventilated rat lungs after lyso-MfEL acetylation [258]. Moreover, Gil et al. also found that lyso-MfEL is more likely to be a fusion component of *M. fermentans* since low-level lysolipids can substantially increase the fusion rate of model membranes and bio-membranes [259].

In addition to the lipids described above, *M. pneumoniae* can produce three glycolipids, five phosphoglycolipids, and six phospholipids [249]. However, the structure of only one glycolipid, *β*-1,6Glc-*β*-Gal-DAG, has been fully characterized; it acts as an important antigen during early infections and participates in the immune response [257]. Chiba et al. have demonstrated that human SP-A and SP-D bind to *M. pneumoniae* through high-affinity interactions with lipids. The major ligand of SP-A is glycolipid, while that of SP-D is phospholipid [265,266]. Interestingly, only SP-A has been shown to be involved in the innate inflammatory response upon *M. pneumoniae* infection [265]. Furthermore, as galactocerebroside has been detected in *M. pneumoniae* [249], it has been postulated that *M. pneumoniae*-triggered Guillain-Barré syndrome, an immune reaction of both the peripheral and central nervous systems against the galactocerebroside, is strongly associated with anti-galactocerebroside IgG [267]. In short, owing to the complexity of the lipid composition and difficult identification methods, the pathogenic roles of lipids in mycoplasmas remain to be further studied.

## MEMBRANE LIPOPROTEINS

Owing to the lack of a cell wall, the mycoplasma cell membrane is composed of a single lipid bilayer with many embedded membrane proteins. These membrane proteins are divided into three categories: integral, peripheral, and lipid-anchored proteins. Most mycoplasmas membrane lipoproteins belong to the lipid-anchored proteins. Like bacterial lipoproteins, mycoplasma lipoproteins contain a conserved S-diacylglyceryl-cysteine residue at the N-terminus. In most mycoplasmas, the NH_2_ in cysteine exists in a free terminal form, yielding a diacylated lipoprotein (in bacterial lipoproteins, there is an additional acyl group in the amino group of the diacylated cysteine residue, i.e. triacylated lipoprotein), which may be one of the reasons why its immune-modulatory mechanism is different from that of bacteria. Mycoplasma membrane lipoproteins are abundantly present in the cell membrane and are pivotal proinflammatory substances of mycoplasmas. In 2018, an elaborate review summarized the effects of membrane lipoproteins on lymphocytes, epithelial cells, neutrophils, and myeloid cells as well as the regulation of the immune system [268]. However, the true function of lipoproteins is far from that; in addition to the function of immunomodulation, membrane lipoproteins are also essential for cell adhesion; biofilm formation (i.e., Vsa protein); cytotoxicity; colonization and invasion of host cells; degradation of host DNA or NETs; and functioning of ABC transporters [57,68,80,86,269]. Christodoulides [268], Shimizu [207] and May [270] have provided complete details regarding these functions. Herein, we will discuss new insights into membrane lipoproteins of pathogenic mycoplasmas.

Ureaplasmas are associated with human genitourinary tract diseases and perinatal diseases. Multiple banded antigen (MBA) is the major surface-exposed lipoprotein of ureaplasmas, which undergoes phase and size variation both *in vivo* and *in vitro*. The characteristics and functions of MBA have been described extensively in other studies [103,139,140]. MBA may be linked to the severity of human placental inflammation *in vivo* and *in vitro* [271], although controversial results exist [272]. The current evidence indicates that a potent host humoral immune response may be a key determinant in explaining adverse pregnancy outcomes and that the degree of MBA/mba variation increases with the duration of gestation *in vivo* [273]. Additionally, Huang and colleagues have indicated that the lipid-associated membrane proteins of *U. parvum* and *U. urealyticum* induce U937 cell cycle arrest in the G1 phase through a p21-dependent but p53-independent pathway [274], suggesting that this signaling pathway is highly correlated with the inflammatory and protective effects in ureaplasmal diseases. Interestingly, a recent report has indicated that the lipid moiety of *M. pneumoniae* lipoprotein is a causative factor of vaccine-enhanced disease, which overcomes the roadblock of vaccine development [14]. This suggests that the development of a vaccine should balance the maintenance of its immune-stimulatory activity and reduce the side effects caused by the lipid moiety. Furthermore, the lipoproteins of *M. pneumoniae* and *M. salivarium* can translocate to the cytosol via an unidentified mechanism, which causes NLRP3 inflammasome activation and IL-1β maturation in macrophages [275]. This illustrates the complexity of the proinflammatory mechanism of lipoproteins.

The interactions between lipoproteins and TLRs have attracted significant attention. Mycoplasma lipoproteins are primarily recognized by TLR2 in cooperation with TLR6. Although some studies have indicated that TLR1 is also involved in this process [276], TLR4 is not involved in lipoprotein recognition. However, Santos-Junior et al. have demonstrated that *U. diversum* lipoproteins interact with TLR4, and then induce higher inflammatory cytokine expression via the NF-кB signaling pathway to evoke an inflammatory response [277]. Nevertheless, we cannot conclude that TLR4 serves as the receptor for *U. diversum*-derived lipoproteins. The endotoxin level in the obtained lipoproteins was not mentioned in their experiment, although an extremely low dose of endotoxin contamination may cause false positive results. At the same time, if any lipids were mingled with the lipoprotein during the extraction procedure, that may also affect the results. Thirdly, there are several factors that affect TLR recognition, including but not limited to the spatial structure or amino acid sequence of some lipoproteins [276]. Santos-Junior’s study was further supported by Shio et al. Both TLR2 and TLR4 interact with HLA-DR and increase the binding and presentation of antigens to T cells following infection with *M. arthritidis* in macrophages [278]. The Peltier group also confirmed that the lipid-associated membrane proteins of *U. urealyticum* can interact with TLR2 and TLR4 to cause inflammation [279]. Therefore, the interaction of ureaplasma with TLR4 warrants further investigation.

The absence of triacylated lipoprotein in mycoplasma has long been a consideration. Although there have been dispersed reports of the possibility that triacylated lipoproteins may exist in some strains, evidence remains lacking. First, neither N-acyltransferase (*Lnt*) nor its homologous gene has been characterized in the mycoplasma genome [280]. Second, when TLR recognizes mycoplasma lipoproteins, not only the lipid moiety, but also the amino acid sequence and spatial structure may affect the recognition of TLRs [281]. Although Shimizu et al. have claimed the existence of triacylated lipoproteins in *M. genitalium* since they were recognized by TLR1 and TLR2, they did not perform any chemical analyses such as the ratio of N-amide and O-ester bonds, and the Edman degradation experiments [282]. Therefore, TLR1 and TLR2 recognition is only a necessary condition for triacylated lipoprotein rather than a sufficient condition. To date, mass spectrometry and nuclear magnetic spectroscopy are more reliable for the identification of triacylated lipoproteinsthan TLR-based estimation. Kurokawa et al. have successfully identified three triacylated lipoproteins in *M. genitalium* (MG_040) and *M. pneumoniae* (MPN052 and MPN415), and one novel peptidyl form of lipoprotein in *M. fermentans* (MBIO_0319 and MBIO_0661) by mass spectrometry [280], suggesting that lipoprotein structures vary among mycoplasmas and even within individual proteins of *M. fermentans*. Given that mycoplasmas lack *Lnt* or its homologues as that in *E. coli*, the existence of the triacylated lipoproteins indicates that the yet to be discovered Lnt enzyme may have distinct enzymatic properties.

With the continuous exploration of mycoplasma virulence molecules, the predominant role of lipoproteins in the proinflammatoryproinflammatory response of mycoplasmas is increasingly fluctuated. The most significant evidence is that the normal flora, which are parasitic in the oral cavity and other parts, also have lipoproteins with a conserved diacylglyceryl structure but usually does not lead to inflammation. In addition, even the same mycoplasma-derived lipoproteins (with the same S-diacylglyceryl-cysteine moiety but different amino acid sequences) display different proinflammatory effects [276]. Future studies remain warranted to understand the pleotropic effects of mycoplasma lipoproteins on the inflammatory response, characterize the potentially active molecule responsible for pathogenic mycoplasmas, demonstrate how the pattern recognition receptor signaling pathway mediates the mycoplasma-associated immune response, and develop new therapeutic strategies against mycoplasma infection.

## SUPERANTIGENS

Superantigens (SAgs) are potent immune-regulatory proteins produced by bacteria, viruses, and *M. arthritidis*. The SAg, which binds to the outside of MHC class II molecules on antigen-presenting cells, is recognized by T cells bearing specific Vβ-chain segments of the T cell receptor (TCR); this interaction does not involve MHC restriction. Thus, SAg can induce a large proportion of peripheral T cells to produce a large panel of cytokines, which has clear implications for pathogenesis [283]. Currently, only *M. arthritidis* has been found to produce SAg. This SAg, MAM, is a haplotype-restricted polyclonal soluble T-cell mitogen and is a small basic, acid-labile protein with a molecular weight ranging from 15 to 30 kDa [284]. In contrast to conventional SAgs, MAM exhibits special characteristics: (I) it preferentially presents T cells through H-2E, H-2A, HLA-DR, and HLA-DQ [285]; (II) the Jb fragment as well as the CDR3 region of the TCR affect the T cell reactivity to MAM [286]; (III) MAM not only binds to TCR Vβ but also interacts with TCR Vα [287]; (IV) formation of a unique MAM/HLA-DR1/hemagglutinin (HA) complex is a prerequisite for T cell activation [288]; (V) TLR2 and TLR4 play vital roles in MAM-induced immune responses [289–291]; and (VI) MAM is the only SAg with potential DNase activity [292].

The mechanism of the interaction between MAM and HLA-DR is becoming increasingly apparent. MAM binds to HLA-DR molecules located in the proximity of the binding groove in a Zn^2+^-dependent manner. The Zn^2+^-binding site at the MAM N-terminus permits dimer formation, MAM homodimerization and MHC class II dimerization/oligomerization. This induces conformational change and exposes MHC class II- and TCR-binding epitopes, resulting in a high affinity combined with HLA-DR, and ultimately induces considerable T cell activation [293]. However, in the three-dimensional structure of the MAM/HLA-DR1/HA complex, no Zn^2+^ has been reported at the interface of the MAM/HLA-DR1 complex or the MAM homodimer [288]. In solution, MAM can form a homodimer at high protein concentrations, irrespective of the presence of Zn^2+^ [294], demonstrating that conformational changes in MAM are independent of Zn^2+^. Subsequently, Li et al. clarified that Zn^2+^ can efficiently induce the dimerization of the HLA-DR1/HA complex, and then synergistically combine with MAM in a 1:1 stoichiometric ratio to form a dimerized MAM/MHC class II complex [295].

Pattern recognition receptors, including TLR2 and TLR4, are also essential to the MAM-triggered immune response apart from MHC class II. This theory is derived primarily from C3H/HeSnJ (TLR2^+^/TLR4^+^) and C3H/HeJ (TLR2^+^/TLR4^−^) mice. After exposure to MAM, C3H/HeSnJ mice induce type 2 cytokines, such as IL-4, IL-6, and IL-10, while C3H/HeJ mice induce type 1 cytokines, such as IL-2, IL-12, TNF-ɑ, and IFN-γ [289]. *In vivo* experiments have demonstrated that, after injection of live *M. arthritidis*, C3H/HeJ mice are more vulnerable to toxic death than C3H/HeSnJ mice. In the same way, the increased sensitivity of C3H/HeJ mice to severe arthritis caused by live *M. arthritidis* is associated with type 1 cytokine profile, whereas, BALB/c mice (TLR4^+^) display a type 2 cytokine profile in response to MAM and develop a mild arthritis caused by *M. arthritidis* [289,296], indicating that MAM differentially regulates the cytokine response by unique interaction with TLRs, and this interaction may be associated with the severity of disease induced by *M. arthritidis*. Mu et al. demonstrated that MAM can directly interact with both TLR2 and TLR4, and that TLR4 signaling may downregulate the MAM/TLR2 inflammatory response in the presence of both receptors [290]. Blockage of C3H/HeN (TLR2^+^/TLR4^+^) mice-derived macrophages by TLR4 antibody induces type 1 cytokine response, which releases high amounts of IL-12p40 in response to MAM. Compared with the wild-type C3H/HeN mice, TLR2-knockout C3H/HeN mice release significantly less IL-12 [290]. These results suggest that TLR2 is involved in the induction of MAM type 1 cytokine production, and that crosstalk between TLR2 and TLR4 affects the nature of the cytokine response pattern. However, Marina et al. showed that MAM cannot bind to TLR2- and TLR4-transfected cells. Co-transfection of TLR2 or TLR4 with HLA-DR significantly increases MAM binding and subsequent T cell activation compared to cells expressing HLA-DR alone. Anti-HLA-DR antibody can eliminate the upregulation of MAM binding and activity in HLA-DR/TLR transfected cells [278], which suggests that co-expression of TLR2 or TLR4 with HLA-DR enhances MAM-induced T cell activation, which is dependent on HLA-DR but completely independent of TLR2 or TLR4.

Moreover, C3H/HeJ upregulates the expression of B7-1 co-stimulatory molecules on peritoneal adherent cells upon MAM stimulation, whereas C3H/HeSnJ downregulates it [291]. Intriguingly, after *in vivo* administration of anti-B7-1 antibody, the type 1 cytokine profile of MAM-injected C3H/HeJ mice changed to type 2 and significantly delayed the lethal toxicity of *M. arthritidis*. On the contrary, type 2 shifted to a type 1 profile and enhanced arthritis in C3H/HeSnJ mice [291]. These results clearly demonstrate the importance of TLR control of B7-1 function in regulating cytokine response and inflammatory disease induced by MAM. Similarly, MAM interacts with TLR2 and TLR4 to differentially modulate IL-17/Th17-related cytokines. In C3H/HeSnJ mice, blockade of TLR4 antibodies decreases the IL-17 and IL-6 production, while TLR2-knockout mice demonstrate increased levels of both cytokines [297], suggesting that TLR4 is required for the IL-17/Th17 cascade. Furthermore, inhibition of B7-1 significantly enhances the MAM-initiated TLR4-dependent IL-17/Th17 cascade [298], thereby aggravating *M. arthritidis*-induced arthritis. Previous studies have shown that the sensitivity of different C3H substrain mice to arthritis is ascribed to the different cytokine profiles induced by MAM [289]. However, in CBA/J and DBA/2 J mice, MAM-induced lethal toxicity is independent of arthritis [299], indicating that MAM is not the primary arthritogenic factor in these strains.

In addition to inducing lymphocyte activation, MAM also induces macrophage activation through TLR2 and TLR4 but does not require CD14 as a coreceptor [290]. The study by Fa´tima et al. showed that MAM-induced NO release is MHC class II-restricted, IFN-γ-, time- and concentration-dependent, and TLR4-independent in C3H/HeJ and C3H/HePas-originated macrophages [296]. Nevertheless, these results are contradictory to those reported by Mu et al., in which stimulation with MAM induces peritoneal or spleen macrophages from C3H/HeJ mice to produce more NO than the co-isogenic C3H/HeSnJ mice [289], demonstrating that the TLR4 signaling pathway may play a fundamental role in initiating the differentiation of cytokine profiles in response to MAM. However, the explanation for these contradictory results is unknown. Additionally, MAM incubated with resident murine peritoneal macrophages can induce PAF production, which regulates NO production by upregulation of iNOS and by controlling the levels of prostaglandin E_2_, which suggests that PAF plays a significant role in the mediation of cell response to MAM [300].

MAM also has potential DNase activity. The seven different amino acids located at the N-terminus of MAM may represent the putative DNase homology region; and substituting one of these amino acids is sufficient to eliminate the enzymatic reaction [292], revealing that the homology region in the MAM N-terminus is important for maintaining its DNase activity. However, this nuclease activity was not substantially reduced in knockout strains [299], indicating that MAM is not the major nuclease in the culture supernatant filtrates.

## CANDIDATE VIRULENCE FACTOR

Christie-Atkins-Munch-Peterson (CAMP)-like factor is a candidate virulence factor for some mycoplasmas. *Streptococcus agalactiae* (group B streptococci [GBS]) can produce CAMP factor, which can enhance the activity of *S. aureus* β-hemolysin to lyse erythrocytes, thereby increasing the hemolytic power at the junction of the two bacteria (CAMP phenomenon) [301]. Kornspan et al. has found that certain mycoplasmas, such as *M. fermentans, M. hominis*, and *M. gallisepticum* can enhance the hemolysis caused by *S. aureus* (positive CAMP phenomenon), while *M. pneumoniae* had a negative CAMP phenomenon. In contrast, *M. capricolum, M. hyorhinis* and Mmm displayed an antagonistic hemolysis with *S. aureus* (reverse CAMP phenomenon). Interestingly, *M. penetrans* showed a positive and reverse CAMP phenomenon [237]. Although an association between the CAMP cohemolysin and virulence has been reported in *Riemerella anatipestifer* [302], the mycoplasmal putative CAMP-like factor remains elusive and merits further investigation.

## FUTURE PERSPECTIVES

With the continuous sequencing of more mycoplasma whole genomes, gene annotation has provided more convenient pathways for elucidating the pathogenic functions of these organisms. Although these hypothetical genes predicted using BLAST or other bioinformatic methods may narrow the scope of research to a certain extent, cognitive limitations exist in comparison to the actual situation *in vivo*. Therefore, the final confirmation of the proposed virulence gene still depends on the knockout of the target gene in living mycoplasmas. Unfortunately, genetic manipulation of mycoplasmas remains challenging. Although novel techniques have been reported to manipulate target genes, the physiological characteristics of mycoplasmas make it impossible to knock out the target genes as easily as in eukaryotic bacteria, such as by RNA interference and CRISPR technology. Hence, several challenges remain before the exploration of mycoplasma-applicable gene editing technology. Our prediction of the molecular structure and function of pathogenic proteins, based on the progress of comparative genomics of homology analysis and crystal analysis technology, has become more accurate, providing favorable conditions for the study of putative genes. In recent years, the characterization of the function and structure of proteins by frozen electron microscopy has become a hot topic, while the high cost makes the general laboratory flinch.

The lipoproteins of mycoplasmas are considered the primary proinflammatory substances [207]. It was originally believed that the lipid structure at the N-terminal of lipoproteins is the structure behind the inflammatory effect and the foundation for TLR2/6 recognition. Increasing evidence shows that in addition to TLR2 and TLR6, TLR1 and TLR4 are both involved in the identification of mycoplasmas [282,303]. TLR1/2 primarily recognizes bacterial triacylated lipoproteins, while TLR4 primarily recognizes LPS [304]. Nevertheless, the presence of N-acyltransferase has not been reported in the mycoplasma genome [280]. Thus, the existence of triacylated lipoproteins in mycoplasmas upon a lipoprotein recognition by TLR1 and TLR2 is not conclusive, unless any direct evidence is obtained by chemical composition identification coupled with mass spectrometry or nuclear magnetic resonance analysis.

Research in the past 20 years has focused on adhesins, lipoproteins, and toxins, while several unaddressed questions regarding the pathogenic role of lipids in the mycoplasma cell membranes remain. A recent report showed that TLR4 can mediate proinflammatory cytokine secretion in TLR2-deficient macrophages upon *M. pneumoniae* infection [303], indicating that TLR4 is a receptor for *M. pneumoniae*. TLR4 recognizes lipid A in LPS of Gram-negative bacteria, which is not found in mycoplasmas. There are abundant lipids on mycoplasma cell membranes, and a considerable proportion of the lipids exist in the form of glycolipids or phosphoglycolipids. Nonetheless, our current understanding of their biological effects is limited, hence lipids might possibly be TLR4 ligands. Our recent data also demonstrate that high concentrations of lipids could trigger the secretion of proinflammatory cytokines in mouse macrophages via TLR4 [305]. Owing to the biochemical particularity of lipids, it is difficult to study their functions through gene knockout as done *in vivo* and *in vitro*, hence resulting in fewer lipids being functionally characterized. In this regard, future studies should focus on identifying mycoplasma functional lipids.
